# RNA-guided nucleases enable a gene drive of insertion sequences in plasmids

**DOI:** 10.1101/2025.02.20.638934

**Published:** 2025-02-21

**Authors:** Kepler S. Mears, Fernando W. Rossine, Natalia Quinones-Olvera, Célia Souque, Michael Baym

**Affiliations:** 1. Department of Biomedical Informatics and Laboratory of Systems Pharmacology, Harvard Medical School, Boston, MA 02115, USA.; 2. Department of Infection Control and Vaccines, Norwegian Institute of Public Health

## Abstract

Mobile genetic elements (MGEs) and the interactions between them are a major source of evolutionary innovation. Insertion sequences, the simplest MGEs usually encoding only the necessary genes for transposition and maintenance, are widespread in bacterial genomes, and are particularly common in plasmids. Plasmids, self-replicating extrachromosomal DNA elements, often exist in multiple copies imparting a stochastic barrier to the fixation of an insertion sequence by limiting the proportion of the plasmid population harboring the IS. In this work we demonstrate that to overcome this, the IS200/605 family of insertion sequences utilizes programmable RNA guided nucleases as gene drive to spread the IS through the plasmid population. TnpB, the likely ancestor of Cas12, records the specific insertion site of the IS in its RNA guide to prevent loss of the IS during transposition. When introduced to a plasmid TnpB will be reprogrammed to target and cleave IS− plasmids, resulting in biased replication of IS+ plasmids. Furthermore, the gene drive activity is critical for the IS to invade high copy plasmid populations. Because TnpB can only be mobilized between microbes on other mobile genetic elements, this advantage to fixing in plasmids may help explain the prevalence of TnpB across the tree of life. More generally, the unique pressures arising from movement between genetic contexts with different multiplicities shapes the evolution of strategies for MGE spread.

## Introduction

Mobile genetic elements interact with each other in distinct ways to modulate horizontal gene transfer as well as promote their own selfish spread^1^. Plasmids, extrachromosomal self-replicating DNA elements, are a major vector of horizontal gene transfer (HGT) in bacteria^2^ and often carry smaller MGE such as transposons^3,4^. Transposons (TNs), short DNA elements capable of excision and insertion within the genome, are frequently found in plasmids^5^. Most transposons rely on transport via other MGEs to move between organisms. In particular Insertion sequences (IS), the most minimal TNs only containing the genes for transposition, strictly cannot move between organisms without a vector and are particularly common in plasmids^3,4,6,7^. The complementary interaction between plasmids and TN/IS sets up a symbiotic relationship, with the TN serving as a vehicle to modify plasmid cargo and the plasmid enabling transfer of TNs, that further drives bacterial genome plasticity.

**Figure 1:**
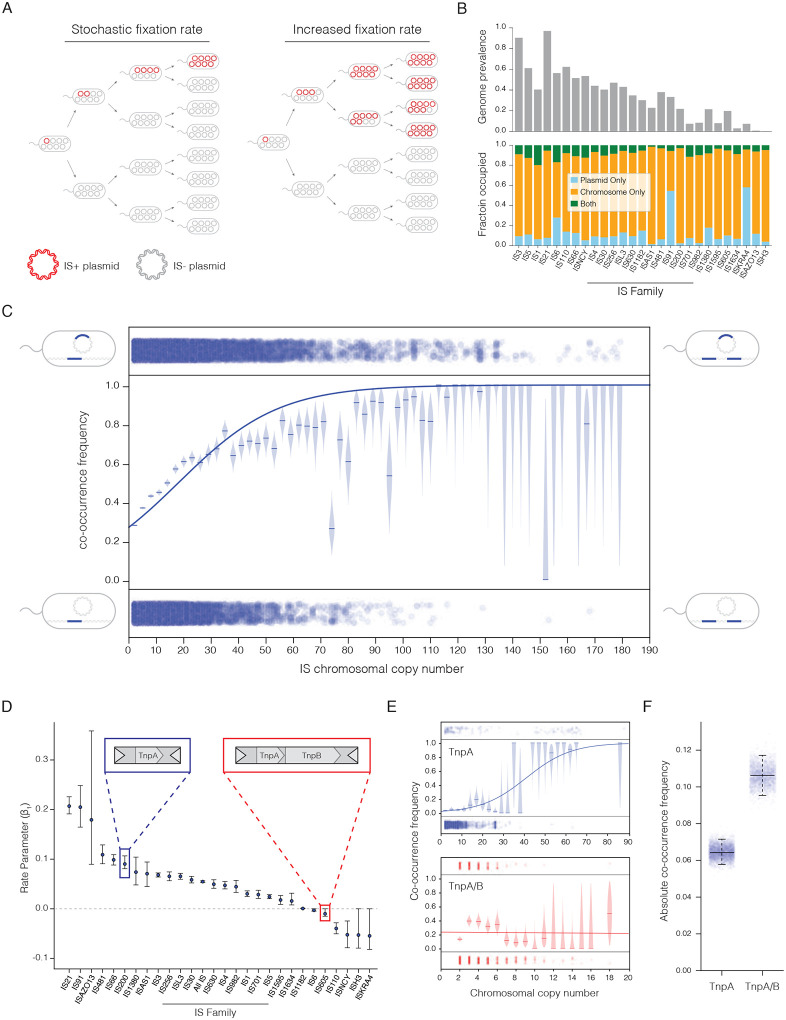
Positive association between chromosomal copy number and plasmid co-occurrence is a potential general mechanism to overcome limits in stochastic fixation for insertion sequences. A) Model of purely stochastic IS fixation in plasmids vs that of an IS with increased fixation rate. IS+ plasmids are indicated in red and IS− in grey B) Prevalence of insertion sequences across genomes in the PLSDB database (top) and distribution across replicon types in genomes (bottom). IS families are ordered from highest overall abundance to lowest as identified in S1B. C) Co-occurrence frequency of insertion sequence in plasmids and chromosomes at given IS chromosomal copy numbers. Dots at the bottom represent an IS found only in the chromosome of a genome while dots on top represent IS that co-occur in the chromosome and plasmid. Binned and calculated frequencies at specified chromosomal copy number are plotted with the expected posterior beta distribution in light blue and medians indicated with a horizontal line and the sigmoid fit determined from the raw data is plotted overtop in solid blue. D) Rate parameters from the sigmoid fit of all IS families analyzed with 95% confidence intervals determined from a chi-squared approximation from a profile likelihood of the rate parameter. E) Individual co-occurrence frequency plots with overlaid sigmoid fit for TnpA only IS in blue, and TnpA/B IS in red, with corresponding binned frequencies, expected posterior and fit. F) Absolute co-occurrence frequency of plasmid presence for TnpA and TnpA/B IS. 95% confidence intervals determined from bootstrapping with each replicate in light blue.

Insertion sequences face a challenge residing on plasmids as they do not inherently provide a selective benefit for the plasmid. Plasmid cargo ideally gives the plasmid an advantage, such as anti-defense factors, or to the host with anti-microbial resistance genes^8–10^. TNs capable of carrying beneficial cargo can bias survival of a TN+ plasmid within the host population. Without such a benefit an IS that inserts into a plasmid will be passed down stochastically to the progeny as the cell divides. Eventually through genetic drift the IS+ plasmid will fix within a subpopulation (each plasmid within the cell being identical i.e. IS+), in a proportion inverse to plasmid copy number (Figure 1A)^11^. The comparably low proportion of IS+ plasmids are liable to be lost if as bacteria inevitably stop dividing or die. It is therefore beneficial for the IS to increase its fixation rate (the fraction of descendants whose plasmids homozygously carry the IS) to increase the spread throughout the population, for example, by carrying a mechanism that would directly increase the proportion of IS+ plasmids.

We propose that the recently described mechanism of the IS200/605 family encoded protein TnpB provides a mechanism to increase the fixation rate of insertion sequences in plasmids. The IS200 family are some of the most minimal insertion sequences containing only a transposase TnpA. The IS605 (including the highly related IS607) family is nearly identical to IS200s except for containing an additional gene TnpB. TnpB is the likely ancestor to Cas12 and similarly is a programmable RNA guided DNA endonuclease and is abundant being found in 30% of bacteria and all three domains of life^12,13,14^. The targeted nuclease activity of TnpB has been characterized as a method for transposon retention in the genome akin to homing endonucleases^15^. IS200/605 elements transpose via a “peel and paste” mechanism triggered during genomic replication, leaving one daughter chromosome devoid of the IS. This non-replicative mechanism leads to a potential decrease of IS prevalence if transposition is not accomplished^16^. TnpB utilizes an RNA guide to record the flanking region of the insertion site of the IS. Like Cas12 TnpB requires an additional Target Adjacent Motif (TAM, like the PAM for CRISPR) to cleave DNA which is the same motif as TnpA’s preferred insertion site. When transposition is triggered the excision junction will bring the TAM and flanking DNA region together to produce the ideal substrate for TnpB. TnpB can then induce a DNA cut and trigger homologous recombination to copy the IS to the now IS− daughter strand to ensure the IS is not lost from the specific insertion site. Functionally, TnpB records the specific prior insertion site of the IS then cleaves any instance of the insertion site it encounters.

In this work we show that the targeted nuclease activity of TnpB functions as a gene drive to spread in plasmids. Gene drives occur in polyploid environments where a DNA element can bias its inheritance by a direct molecular mechanism. TnpB accomplishes this through its programmable RNA guided nuclease activity that can be used to selectively target plasmids that do not contain the IS. Our results characterize a novel gene drive and explain a specific method used by an IS to disseminate throughout bacteria. Additionally, we further deepen the understanding of how TnpB has become so prevalent in bacterial genomes.

## Results

### Presence of TnpB breaks the positive association between chromosomal copy number and plasmid carriage of IS

We hypothesized that a general mechanism to increase fixation rate of IS could be achieved through repeated attempts of an IS inserting into a plasmid from the chromosome. This could be accomplished by increasing the chromosomal copy number of the IS enabling more transposition events. We searched 19658 complete contiguous genomes from the PLSDB database of plasmids for insertion sequences with HMMER and ISEScan to identify all IS elements, and determined their location (chromosome or plasmid)^17,18^ (Supplemental Figure 1A). We found IS3 to be the most abundant IS, (Supplemental Figure 1B) while IS21 was the most commonly occurring in genomes with over 90% of all genomes containing one or more instances (Figure 1B). The majority of IS elements were identified solely in the chromosome, aside from IS91 and ISKRA4 which were predominantly in plasmids (Figure 1B). IS elements were commonly found in plasmids with 32641 plasmids out of 59895 total containing one or more IS elements.

We observe that overall, presence of an IS in both the chromosome and plasmid (co-occurrence) is positively correlated with chromosomal copy number of the IS (Figure 1C). We filtered our dataset for genomes with a chromosomal IS and determined the number of copies in the chromosome. We then identified those that contained the IS only in the chromosome, and those that co-occurred in plasmid (1C, dots bottom and top respectively). From this data we calculated the co-occurrence frequencies at all chromosomal copy numbers (Figure 1C). The resulting co-occurrence frequencies indeed increased with chromosomal copy number and the trend modeled by a sigmoid function (eq 1). The rate parameter, β_1_, from the sigmoid function provides a summarization of the sigmoid fit with a β_1_<0 indicating negative association with chromosomal copy number and β_1_>0 a positive association. As expected from the overall trend, the majority of IS families had positive rate parameters (Figure 1D) which could also be seen in their individual sigmoid fits (Supplemental Figure 1C) with a handful of outliers having β_1_<0 or β_1_≈0. A few outliers, notably ISH3 and ISKRA4, are likely due to the minimal data and abundance of these IS preventing an association being derived with confidence (Supplemental Figure 1C).


Eq 1 $$p\left(x\right)=\frac{1}{1+e^{-\left(\beta_{0}+\beta_{1}x\right)}}$$

We noticed a striking difference between the highly related IS200 and 605 families (Which for clarity we differentiate as TnpA and TnpA/B IS respectively). The IS200 subfamily followed the general trend with β_1_<0 while IS605 did not with a β_1_≈0, despite being nearly identical aside from the addition of TnpB. Indeed, the difference was apparent in the respective sigmoid fits for each with TnpA having a significantly positive association with chromosomal copy number and TnpA/B having no obvious association (Figure 1E). Specifically at low chromosomal copy number TnpA/B had a much higher co-occurrence frequency than TnpA, implying that TnpB increases fixation rate at low chromosomal copy number (Supplemental Figure 1D). To measure the overall impact of TnpB the absolute co-occurrence was determined by looking at each possible IS and plasmid pairing within each genome. TnpA only IS had an absolute co-occurrence frequency of ~0.6 while the addition of TnpB in TnpA/B IS was nearly doubled the co-occurrence ~0.11 (Figure 1F). Taken together our results indicate that the addition of TnpB fundamentally changes the interaction between the IS and plasmids, leading to a greater co-occurrence of IS in plasmids.

### TnpB functions as a gene drive to allow IS+ plasmids to overcome stochastic fixation

**Figure 2:**
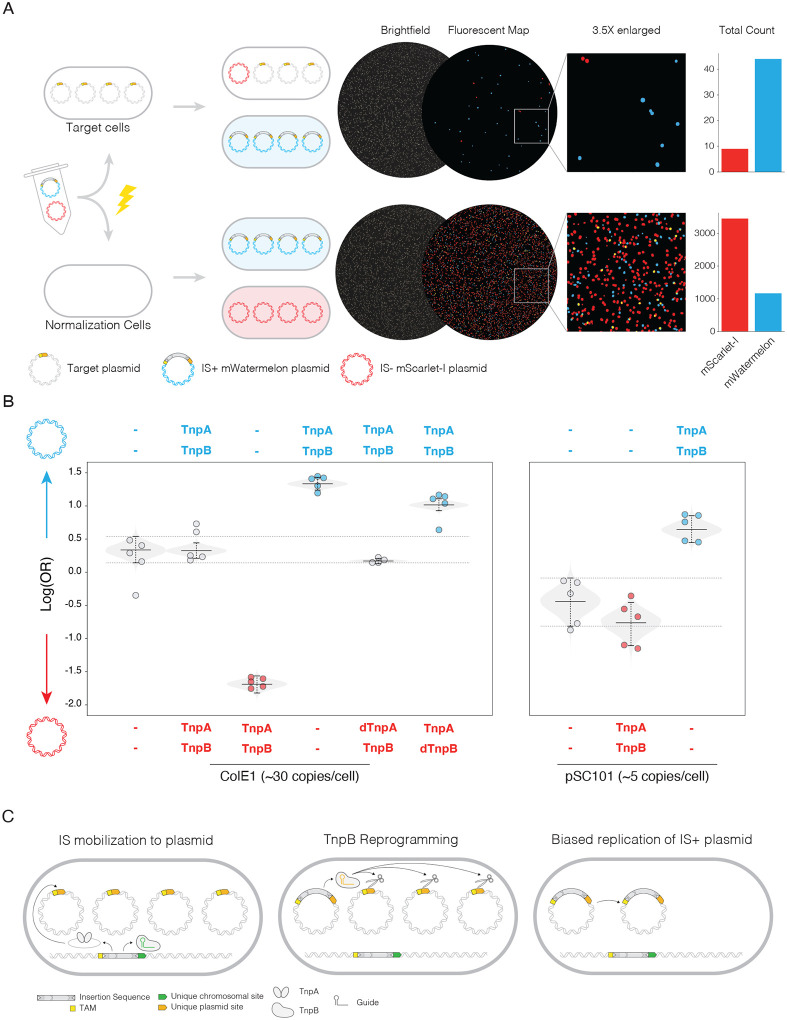
TnpB enables IS+ plasmids to more efficiently displace IS- plasmids A) Experimental workflow for plasmid displacement assay and example replicates. Fluorescent maps are representations of the fluorescence signal that have been enhanced for visualization B) Log-odds ratio of colony counts for ColE1 plasmid displacement assays. mWatermelon plasmid variants are labeled on top and mScarlet-I on bottom. Individual replicates are plotted with the expected posterior in light grey and 95% confidence intervals. Dotted line is 95% confidence interval from the empty-empty plasmid condition. Right box contains selected data for pSC101 origins. C) Proposed gene drive model for the advantage of IS+ plasmids. Insertion sites/TAM is indicated in yellow with the genomic flanking region in green and plasmid in orange.

To test our observation that TnpB provides an advantage to plasmids we experimentally measured whether a TnpB+ plasmid has an advantage in replacing copies of itself lacking TnpB. We electroporated a mixture of two plasmids labeled with related fluorescent proteins mScarlet-I and mWatermelon into a test cell line containing a non-fluorescent target plasmid (Figure 2A) with a validated insertion/excision site from a TnpA/B IS^14,19^. In parallel we electroporated the plasmid mix into plasmid empty cells as a normalization control. From fluorescent images we compared the ratio of fluorescent colonies in the target cell to the normalization plates and calculated the log odds ratio reflective of the ability of each test plasmid to replace the target plasmid, which we defined as positive if the blue plasmid had an advantage and negative if red (Supplemental Figure 2 A/B). A plasmid containing an IS with a matched insertion site to the target plasmid mimics an insertion event, and based on our bioinformatic results would be expected to have an advantage in competition compared to plasmids that do not.

We first measured the effect of TnpB in high copy ColE1 plasmids. Plasmids with a TnpB+ IS displayed a significant advantage displacing the target plasmid compared to IS− plasmids regardless of the dye orientation (Figure 2B). Equally matched plasmids, either both containing the IS or both empty, produced near zero log odds ratio indicating neither plasmid had an advantage, and the assay was functioning as expected (Figure 2B). Competition of an IS+ plasmid and a plasmid with a catalytically inactivated TnpA also produced a near zero log-odds and thus TnpA was not responsible for the IS+ plasmid’s advantage.

Catalytic inactivation of TnpB’s RuvC domain eliminated the advantage of an IS+ plasmid. Competition of an IS+ plasmid with an IS+ plasmid harboring dTnpB resulted in a log-odds favoring the unmodified TnpB, indicating inactivation of TnpB rendered a comparable advantage as an IS− plasmid. As the RuvC domain is required for DNA cleavage it is the nuclease activity of TnpB that is likely driving the observed difference.

This advantage provided by TnpB is held over different multicopy plasmid types. We repeated the experiment with the lower copy pSC101 origin, estimated 5 copies per cell compared to ColE1’s 30 and produced similar results (Figure 2B). A lower copy number results in an increased effect of stochastic segregation, leading to an increased fixation rate for any novel plasmid. Thus, any further increase in fixation rate given to a plasmid will be less impactful. The magnitude of advantage with pSC101 origins was much smaller, confirming that the advantage is sensitive to plasmid copy number and furthermore not specific to ColE1.

As further support that the TnpB+ plasmids were in fact actively replacing the target plasmid population we compared the colony luminescence (Supplemental Figure 2C). Brightness was comparable between plasmids with the same fluorophore in the normalization plates regardless of IS presence in the plasmid, suggesting the IS does not impart any effect on plasmid copy number, which could account for the advantage. While the mWatermelon channel was generally brighter, this did not affect the results regardless of threshold (Supp Fig 2D). In the target plates IS+ plasmids resulted in brighter colonies than IS− indicating the proportion of IS+ was increasing within the cells (Supplemental Figure 2C).

We conclude that TnpB’s endonuclease activity explains the observed advantage an IS provides the plasmid. Furthermore, we hypothesize that the programmability of TnpB’s guide enables a specific mechanism of the IS to fix within the plasmid population. When the IS transposes into a plasmid the newly inserted TnpB will be reprogrammed to the unique plasmid insertion sites. Since plasmids are initially genetically identical, this insertion site will exist on all other plasmid copies (Figure 2C), which is the ideal substrate for TnpB cleavage. Cleavage by TnpB will result in destruction of a plasmid and bias the replication of plasmids containing the IS, regardless of any fitness disadvantage the IS may apply to the plasmid.

The experiment described here was designed to mimic a transposition event with a target plasmid that specifically matches the TnpB guide in the IS. This design was to isolate the effect of TnpB but does not incorporate reprogramming of TnpB’s guide nor the transposition activity of TnpA. To test whether this ability is enabled by TnpB’s programmable RNA guide, we sought to test whether this effect held when an existing plasmid gained a new IS.

### Fixation of novel transpositions into plasmids is enabled by TnpB in a copy number-dependent manner

**Figure 3:**
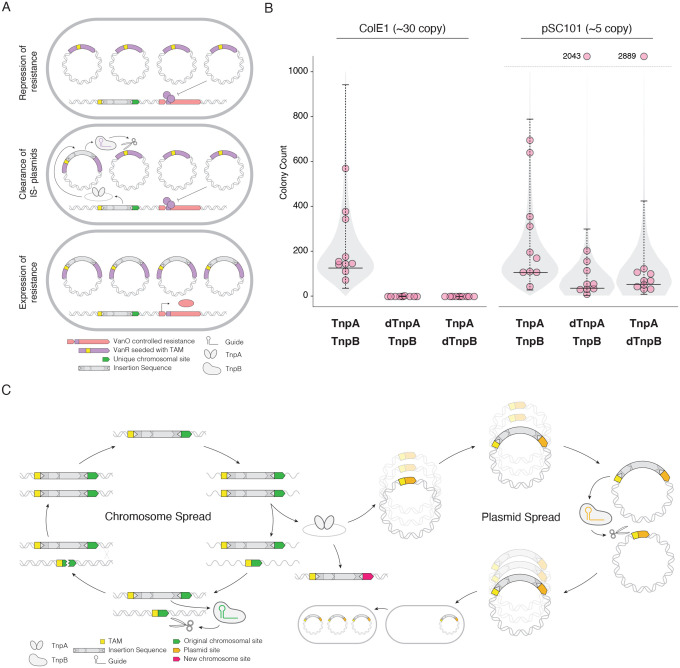
TnpA/B IS are captured at higher rates than TnpA IS in a plasmid targeted transposon trap A) Experimental workflow for transposon trap assay. Vanillin repressor in purple, resistance cassettes in pink, transposon in grey. Yellow indicates potential insertion sites for TnpA. B) colony counting results for insertion sequence variants and target plasmid pairs (indicated on bottom). 95% confidence intervals are indicated with bars around the median from 10 individual replicates as determined from an expectation maximization of the Luria-Delbrück distribution C) Extended model of TnpB IS mobility. TnpB enables genomic spread through retention at specific sites and plasmid spread through the gene drive described in this study, and eventual organism spread through HGT via the plasmid.

To measure the effect of novel insertion events in plasmids, we implemented a transposon trap like those designed to discover novel active transposons^20^. We placed a TnpA/B IS in the chromosome along with a resistance cassette under the control of the vanillin operator. We then introduced a target plasmid expressing the vanillin repressor into the cells, resulting in repression of the chromosomal resistance gene and preventing survival on selective media (Figure 3A). We further seeded the coding region of the repressor with potential TnpA insertion sites, i.e. TAMs, setting up a circuit where transposition by TnpA would disrupt the repressor coding region, rendering repressor the non-functional (Figure 3A). Critically the circuit requires that the disrupted repressor plasmid fixes in the, as one intact repressor coding region is sufficient to produce enough repressor to inhibit transcription of the resistance gene. Therefore, this system quantifies the rate of transposition-and-fixation events.

An active TnpA/B IS in the chromosome produced significant survival after incubation and selection with a ColE1 target plasmid (Figure 3B, left). The result of 10 individual replicates followed an expected long tailed Luria-Delbrück distribution, common to similar mutation accumulation experiments. Catalytic inactivation of TnpA resulted in almost no survival, which was expected as the inability to transpose would prevent disruption of the repressor and resistance expression (Figure 3B). These two results indicate the circuit works effectively and any difference in survival would be indicative of an impaired ability to disrupt the plasmid population.

Inactivation of TnpB resulted in no detectable survival in the ColE1 condition, demonstrating that the programmable nuclease ability of TnpB was critical to fix the plasmid population. We repeated the experiment with the lower copy pSC101 origin in the target plasmid and found a similar level of survival with an active TnpA/B IS compared to ColE1. However, in the dTnpA and dTnpB conditions we observed detectable levels of survival with some replicates within the range of the fully active condition (Figure 3B, right). As with the superinfection experiment, we expected the lower copy number to decrease the advantage of TnpB. A lower copy number allows for a greater stochastic advantage of a novel plasmid, whether generated from an insertion event or mutation. Some survival in the dTnpA and dTnpB conditions was likely due to background mutations that we could not eliminate. There was a slight increase in survival for the dTnpB condition compared to dTnpA, which we expected to see as transposition is still active and able to disrupt the repressor. The pSC101 result demonstrates that the effect of TnpB is magnified with the increased copy number of the target plasmid. Taken together, the transposon trap reveals TnpB improves fixation of newly introduced ISs in multicopy plasmids, to the point of being effectively required for higher copy plasmids.

## Discussion

From an analysis of IS replicon frequency we identified a distinct relationship between IS elements with TnpB and plasmids. We find that IS elements with TnpB grant plasmids an advantage in displacing target plasmid populations and that TnpB affects this phenotype. Specifically, TnpB’s programmable nuclease activity enables displacement and invasion of non-IS plasmids population. Moreover, the copy number of the target plasmid modulates the effect with TnpB being of greater importance with increased copy number. We propose an additional role of TnpB in the life cycle of IS200/605 elements, functioning as a gene drive to spread the IS in plasmid in addition to ensuring retention during mobilization (Figure 3C).

Gene drives require polyploid environments which are typically described in cases with multiple chromosome copies like in eukaryotes. Our work showcases how even the simplest instances of polyploidy - plasmids - are subject to gene drives. Traditional chromosomal gene drives are also constrained by the number of chromosome copies. The existence of plasmids in different copy numbers allows us to interrogate an unexplored dimension of gene drives, the number of targets. We find that the higher the copy number, the greater effect the drive has on gene spread. Furthermore, gene spread in bacteria is typically associated with the fitness benefit given to the host organism where in the case of TnpB the drive is neutral with regards to the host. Altogether, our data demonstrates a unique case of gene dissemination, where the gene drive is phenotypically neutral, and the strength is modulated by copy number.

Despite being a silent phenotype, the acquisition of IS by plasmids is a critical process underlying plasmid evolution and enabling the flexibility of the bacterial pangenome. Insertion sequences serve as recombination hot-spots to allow mobilization of normally immobile elements, enable flexible adaptation of bacteria to environments, and allow plasmid reorganization of gene through composite transposons^21–23^. The ability for TnpB to expedite the spread of IS in plasmids is thus a potentially powerful mechanism of plasmid diversification.

TnpB’s unique interaction with plasmids provides insights into the spread of TnpB itself. TnpB is an incredibly widespread protein in bacteria, occurring in roughly 30% of all genomes despite having low predicted mobility^12^. More impressive is TnpB has been frequently domesticated for other functions, most noticeably CRISPR where TnpB likely evolved into the Cas12 effector. Indeed TnpB’s function in the plasmid gene drive we describe is analogous to the function of CRISPR protection from plasmids, and many Cas12 spacers are predicted to target plasmids^12^. The evolutionary function of TnpB IS gene drives in plasmids helps explain the widespread presence of TnpB and plausibly aided the development of type V CRISPR systems.

While we demonstrate a clear phenotype both computationally and experimentally there are a few limitations of our approach. There are comparably few publicly available contiguous bacterial genomes and the majority are *E coli* subjecting our analysis of the PLSDB database to a bias not reflective of the true underlying prevalence of insertion sequences. Single cuts are usually enough to eliminate plasmids; however, it is possible that the IS elements are spreading through DNA damage induced recombination in plasmids naturally, akin to homing endonucleases. Lastly our transposon trap constitutively expresses the repressor allowing effects from protein production and dilution. Since only a few copies of repressor protein are needed to prevent resistance, we could be undercounting survival and slight effects, particularly with higher copy plasmids that produce more repressor protein. Regardless of the exact details of the underlying mechanism, our results are unambiguous that TnpB is enabling a significant advantage to IS entering plasmid populations.

The IS200/605 family stood out due to the recent body of work on TnpB’s mechanism, but we suspect there are likely other methods of plasmid-IS synergy and inter MGE gene drives. Our bioinformatic results suggest other insertion sequences have distinct mechanisms of fixing in plasmid populations. Given the intense selective advantage of a gene drive for spread on plasmids, we expect this to be a major driver in the evolution of the diverse mechanisms that regulate the transposition and retention of insertion sequences.

## Methods

### Curation of PLSDB database

The PLSDB meta-archive and triangle file was downloaded from https://ccbmicrobe.cs.uni-saarland.de/plsdb2025/download then unique assembly NCBI accession numbers collected. Assembly genbank and FASTA files were downloaded from NCBI and replicon accession numbers and identities added to our database metadata. Assemblies that did not have genbank annotations were annotated with Prokka. Highly similar plasmids were collected into families from PLSDB’s hash table to identify redundant sequences.

### Insertion sequence identification

ISEScan was run on each assembly fasta and the output collected into a single metadata file with a custom python script. IS presence on plasmids vs chromosomes was determined from the ISEScan results and PLSDB metadata. The IS family and plasmid/chromosomal copy number was determined from the ISEScan output^18^.

To find specific IS200, IS605 and IS607 subgroups a HMMER search was performed separately on the PLSDB genbank files using models NF033573.1 and NF033518.0 for TnpA and models TIGR01766.2, NF040570.1, NF038281.1, NF038280.1, PF07282.15, NF040563.1 for TnpB/IscB. The final HMMER output was collected into a single file and pairs of TnpA and TnpB identified as those being within 1000 kb of each other with a custom script. An E-value threshold of 10E-25 was used to filter the final hits and presence on plasmids and chromosomes identified. A pairwise blast or MMSeqs2 were utilized to identify clusters (sequence identity >90%). Clusters were then used to determine the chromosomal copy number.

To determine co-occurrence frequency genomes were filtered for IS chromosomal copy number greater than two to ensure the IS was mobile. Each cluster in each genome was scored as 0 in the cluster only occurring in the chromosome and 1 if in the chromosome and plasmid. A sigmoid was fit to the data and co-occurrence frequency calculated through aggregating and averaging all points at a particular copy number.

### Superinfection assay

RecA deficient TOP10 cells were transformed with a target plasmid containing a dead fluorophore with kanamycin resistance and a TnpB target site for the IsDra2 insertion sequence^14^. Electrocompetent cells were generated by growing to OD = 0.8 at 30 °C, centrifuged at 2500xg, washed with ice cold water before being resuspended in 25% glycerol at 100X concentration. Plasmids 100 ng of each of two plasmids of interest were mixed and diluted to 5uL which were then electroporated into 50uL of target plasmid cells and empty standard TOP10 cells. TOP10 were incubated at 37 °C for 1 hour prior to plating while the target plasmid cells were plated immediately. The same kanamycin resistance as the target plasmid was used in the plasmids of interest, requiring high density plating to obtain enough colonies. This was done to capture fluorescent dynamics, in addition to preventing selection for electroporation events as opposed to fixation events.

A dilution series of cells were plated on 2% Lb agar with 1% synthetic iron oxide and kanamycin. 0.5mL of each dilution was mixed with 5mL of 50C 0.5% LB Agar then plated. After allowing the top agar to solidify a second layer of top agar was added. Plates were incubated at 37 °C overnight. The following day the top agar layer was dried at 37 °C until collapsed to allow oxygen mediated maturation of the fluorophores. Plates were incubated at 4 °C overnight then imaged the next day.

Final images were taken and analyzed with a custom python script. Images were first intensity normalized and a mask to eliminate the plastic plate boundary that produced significant autofluorescence in the green channel. A minimum threshold was estimated from the pixel distribution and a watershed segmentation algorithm applied to identify and count colonies. Additional thresholds based on size and intensity were applied then the overall intensity value adjusted to match the green and red channels. Re-coloring and dilation were applied separately for visualization.

### Fluorescent imaging

To ensure high quality images synthetic black iron oxide was added to all LB agar plates to produce a black background. Plates were imaged with a custom fluorescent plate imager. Plates are illuminated by colored LEDs with excitation filters (EX), the emitted light passes through emission filters (EM), and images are taken on a Canon EOS camera. The red channel pairs 567 nm LED with 562 nm EX and 641/75 nm EM filters, the green channel pairs 490–515 nm LED with 494 nm EX and 540/50 nm EM filters, and the blue channel pairs 448 nm LED with 438 nm EX and 483/31 nm EM filters. Exposure times varied between 0.5s and 8s depending on the plate and intensity of fluorescent signal.

Channel intensity images were taken of a white background and no filter for exposure times of 1/20–1/30s to normalize spatial differences in fluorescence. Camera settings were kept at an aperture of 10 and ISO of 200. Final images were intensity normalized using these background images.

### Transposon trap

A chloramphenicol resistance cassette and the red reporter mScarlet-I, both with expression controlled by the vanillin operator, were introduced in the LacI locus of the genome of transposon and insertion sequence devoid MDS42 cells^24^ with lambda red recombinase. The recently described *Clostridium botulinum* IS607 was introduced into the chromosome (at the araBAD locus) due to its characterized highly active TnpA and TnpB^25^.

We created an optimized coding sequence for the vanillin repressor (VanR) that was saturated with TAMs for IS607 (VanTAM). Plasmids (PBR origin ~20 copies per cell and PSC origin ~5 copies per cell) carrying VanTAM, the green reporter protein mWatermelon, and a Kan resistance cassette were transformed into these cells. A dilution series was performed and inoculated into 1mL in 96 well-plates. Cultures were grown to saturation for 18 hours. Dilutions in which each well was inoculated with on average at most a single cell were selected by checking that most wells were empty after the growth period. The content of ten wells that had cultures grow to saturation was mixed into 3mL of 0.5% agar and overlaid on dishes containing kanamycin and chloramphenicol. In this way, only cells that retained the plasmid but disrupted the repressor could form colonies. Moreover, the green marker allowed us to assess that the plasmid was retained, and the red marker functioned as an independent marker for the disruption of the repressor gene. Plate imaging was performed as in the other assays.

Colony counts were determined with a custom python script and 95% confidence intervals calculated from an expectation maximization of the Luria-Delbruck distribution.

### Code availability

Jupyter notebooks to analyze the data and generate figures can be found in the github repository https://github.com/keplermears-hms/TnpB-gene-drive/tree/main along with bash scripts and snakemake pipelines to generate the data.

## Supplementary Material

Supplement 1 Supplemental Figure 1: Identification and analysis of insertion sequence families in the PLSDB database
A) Bioinformatic pipeline for the curation of the PLSDB replicons and identification of insertion sequences. B) Total hit results of each IS family from the analysis in figure 1. C) Overlaid co-occurrence frequencies of TnpA and TnpA/B IS for chromosomal copy numbers 2–10. D) Sigmoid fits for all IS analyzed in this study and used to generate rate parameters in figure 1D.

Supplement 2 Supplemental Figure 2: Imaging pipeline for superinfection assays
A) Image workflow for colony counting. Overlapping colonies are only removed for test plates. B) image workflow for visualization. Binary threshold is imposed for colonies and the test colonies are dilated. C) Aggregate results prior to calculation of the log odds ratio. Plasmid conditions shown on the right in order top to bottom: both with IS, red with IS blue empty, blue with IS red empty, both empty. D) Threshold sensitivity analysis for the conditions in C for individual channel brightness thresholding and overall brightness and size thresholding. Tiling threshold difference in intensity between the fluorescence channels, and in intensity and colony size resulted in minimal deviation of the final log-odds ratio. There was slight sensitivity when competing two empty plasmids, where we saw a bias towards mWatermelon which was mirrored in our results. We attributed this bias to watermelon generally being a brighter fluorophore.

## References

[1] Horne T., Orr V. T. & Hall J. P. How do interactions between mobile genetic elements affect horizontal gene transfer? Curr. Opin. Microbiol. 73, 102282 (2023). 36863168 10.1016/j.mib.2023.102282

[2] Rodríguez-Beltrán J., DelaFuente J., León-Sampedro R., MacLean R. C. & San Millán Á. Beyond horizontal gene transfer: the role of plasmids in bacterial evolution. Nat. Rev. Microbiol. 19, 347–359 (2021). 33469168 10.1038/s41579-020-00497-1

[3] Siguier P., Gourbeyre E. & Chandler M. Bacterial insertion sequences: their genomic impact and diversity. FEMS Microbiol. Rev. 38, 865–891 (2014). 24499397 10.1111/1574-6976.12067 PMC7190074

[4] Siguier P., Gourbeyre E., Varani A., Ton-Hoang B. & Chandler M. Everyman’s guide to bacterial insertion sequences. in Mobile DNA III 555–590 (ASM Press, Washington, DC, USA, 2015). 10.1128/microbiolspec.MDNA3-0030-2014 26104715

[5] Babakhani S. & Oloomi M. Transposons: the agents of antibiotic resistance in bacteria. J. Basic Microbiol. 58, 905–917 (2018). 30113080 10.1002/jobm.201800204

[6] Leclercq S., Gilbert C. & Cordaux R. Cargo capacity of phages and plasmids and other factors influencing horizontal transfers of prokaryote transposable elements. Mob. Genet. Elements 2, 115–118 (2012). 22934247 10.4161/mge.20352 PMC3429520

[7] Che Y. Conjugative plasmids interact with insertion sequences to shape the horizontal transfer of antimicrobial resistance genes. Proc. Natl. Acad. Sci. U. S. A. 118, e2008731118 (2021). 33526659 10.1073/pnas.2008731118 PMC8017928

[8] Xue W., Hong J. & Wang T. The evolutionary landscape of prokaryotic chromosome/plasmid balance. Commun. Biol. 7, 1434 (2024). 39496780 10.1038/s42003-024-07167-5 PMC11535066

[9] Castañeda-Barba S., Top E. M. & Stalder T. Plasmids, a molecular cornerstone of antimicrobial resistance in the One Health era. Nat. Rev. Microbiol. 22, 18–32 (2024). 37430173 10.1038/s41579-023-00926-x PMC12440250

[10] Samuel B., Mittelman K., Croitoru S. Y., Ben Haim M. & Burstein D. Diverse anti defence systems are encoded in the leading region of plasmids. Nature 635, 186–192 (2024). 39385022 10.1038/s41586-024-07994-w PMC11541004

[11] Rossine F., Sanchez C., Eaton D., Paulsson J. & Baym M. Intracellular competition shapes plasmid population dynamics. bioRxiv (2025) doi:10.1101/2025.02.19.639193. PMC13316098 41264676

[12] Altae-Tran H. Diversity, evolution, and classification of the RNA-guided nucleases TnpB and Cas12. Proc. Natl. Acad. Sci. U. S. A. 120, e2308224120 (2023). 37983496 10.1073/pnas.2308224120 PMC10691335

[13] Saito M. Fanzor is a eukaryotic programmable RNA-guided endonuclease. Nature 620, 660–668 (2023). 37380027 10.1038/s41586-023-06356-2 PMC10432273

[14] Karvelis T. Transposon-associated TnpB is a programmable RNA-guided DNA endonuclease. Nature (2021) doi:10.1038/s41586-021-04058-1. PMC8612924 34619744

[15] Meers C. Transposon-encoded nucleases use guide RNAs to promote their selfish spread. Nature 622, 863–871 (2023). 37758954 10.1038/s41586-023-06597-1 PMC11758364

[16] He S. The IS200/IS605 family and “Peel and Paste” single-strand transposition mechanism. Microbiol. Spectr. 3, (2015). 10.1128/microbiolspec.MDNA3-0039-2014 26350330

[17] Schmartz G. P. PLSDB: advancing a comprehensive database of bacterial plasmids. Nucleic Acids Res. 50, D273–D278 (2022). 34850116 10.1093/nar/gkab1111 PMC8728149

[18] Xie Z. & Tang H. ISEScan: automated identification of insertion sequence elements in prokaryotic genomes. Bioinformatics 33, 3340–3347 (2017). 29077810 10.1093/bioinformatics/btx433

[19] Gohil K., Wu S.-Y., Takahashi-Yamashiro K., Shen Y. & Campbell R. E. Biosensor optimization using a FRET pair based on mScarlet red fluorescent protein and an mScarlet-derived green fluorescent protein. bioRxiv (2022) doi:10.1101/2022.06.20.496847. 36693235

[20] Tansirichaiya S., Moyo S. J., Al-Haroni M. & Roberts A. P. Capture of a novel, antibiotic resistance encoding, mobile genetic element from Escherichia coli using a new entrapment vector. J. Appl. Microbiol. 130, 832–842 (2021). 32881179 10.1111/jam.14837

[21] Chen K., Xie M., Chan E. W.-C. & Chen S. Delineation of ISEcp1 and IS26-mediated Plasmid fusion processes by MinION single-molecule long-read sequencing. Front. Microbiol. 12, 796715 (2021). 35197941 10.3389/fmicb.2021.796715 PMC8859459

[22] Sastre-Dominguez J. Plasmid-encoded insertion sequences promote rapid adaptation in clinical enterobacteria. Nat. Ecol. Evol. (2024) doi:10.1038/s41559-024-02523-4. PMC7616626 39198572

[23] He S. Insertion sequence IS26 reorganizes plasmids in clinically isolated multidrug-resistant bacteria by replicative transposition. MBio 6, e00762 (2015). 26060276 10.1128/mBio.00762-15 PMC4471558

[24] Nyerges A. A swapped genetic code prevents viral infections and gene transfer. Nature 615, 720–727 (2023). 36922599 10.1038/s41586-023-05824-z PMC10151025

[25] Žedaveinytė R. Antagonistic conflict between transposon-encoded introns and guide RNAs. Science 385, eadm8189 (2024). 38991068 10.1126/science.adm8189 PMC11758368

