# Supplementary figures and images for: RNA-guided nucleases enable a gene drive of insertion sequences in plasmids
Source: bioRxiv. 2025 Feb 21:2025.02.20.638934. Preprint. [Version 1] doi: 10.1101/2025.02.20.638934 (PMC11870625; doi:10.1101/2025.02.20.638934)

A

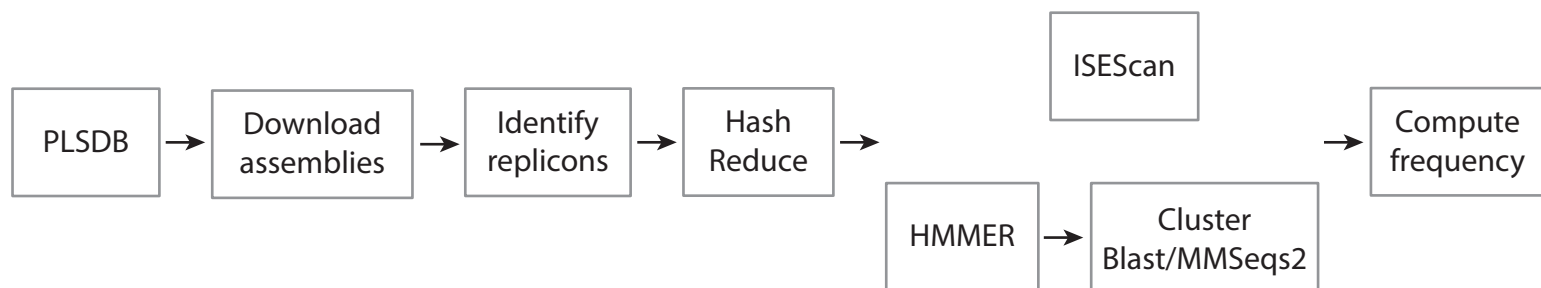

B

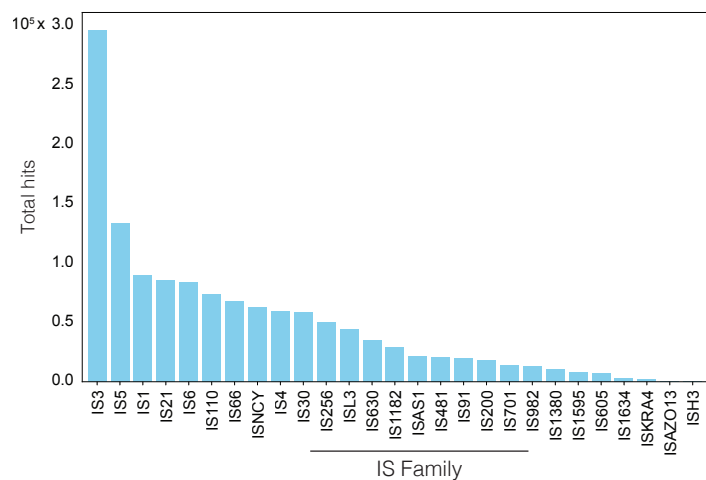

D

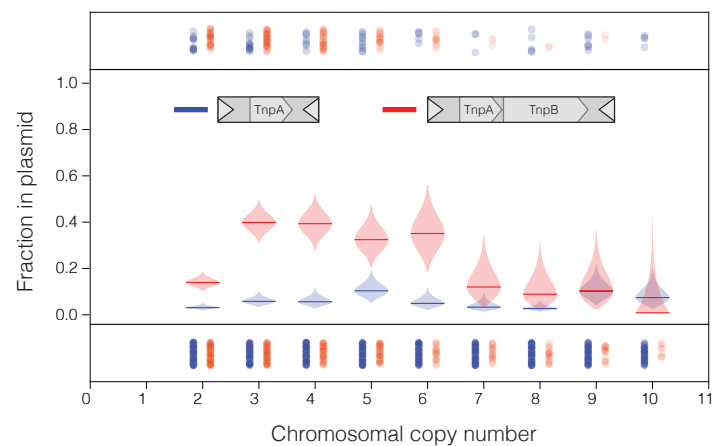

C

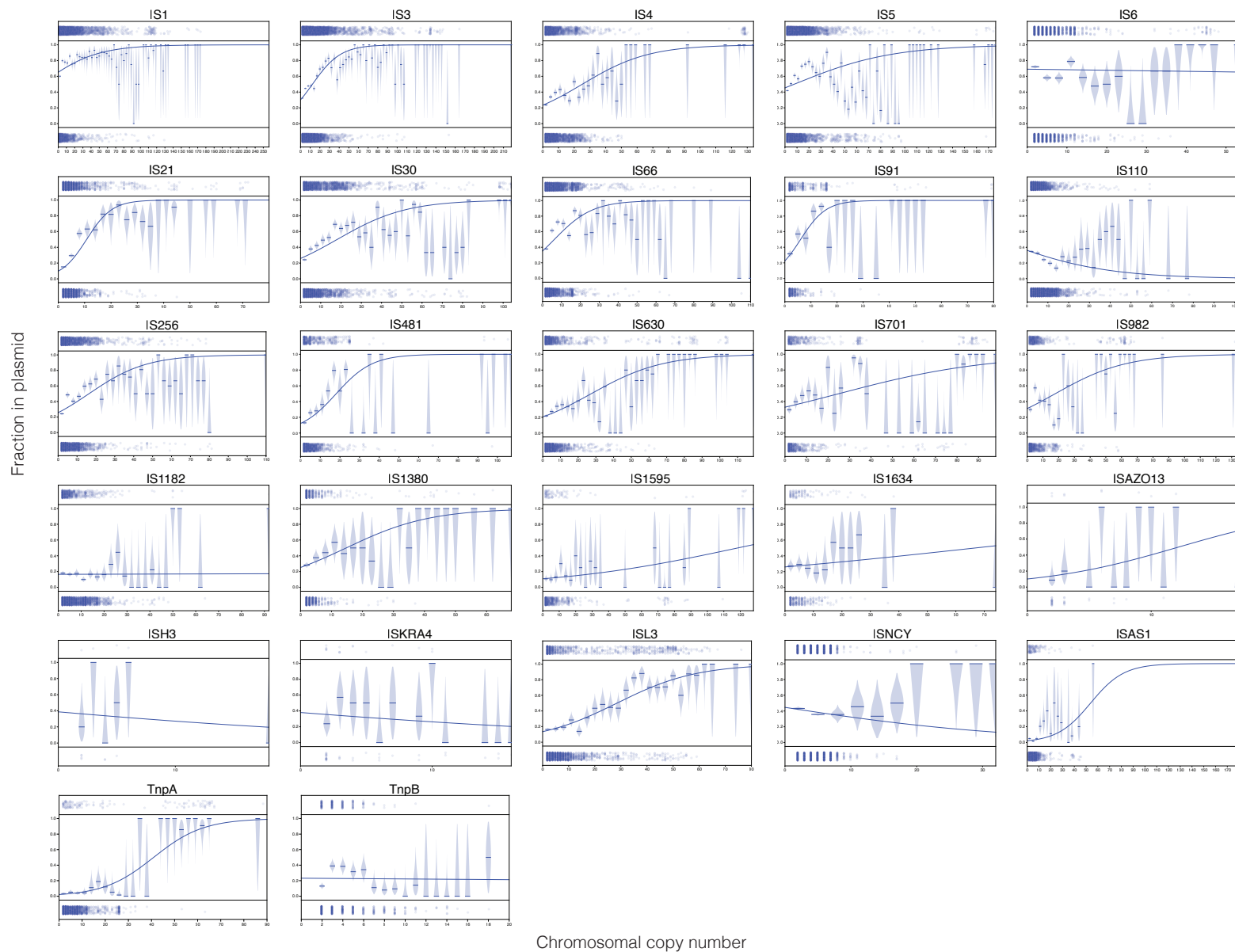

Supplement: Supplement 1 — Supplemental Figure 1: Identification and analysis of insertion sequence families in the PLSDB database A) Bioinformatic pipeline for the curation of the PLSDB replicons and identification of insertion sequences. B) Total hit results of each IS family from the analysis in figure 1. C) Overlaid co-occurrence frequencies of TnpA and TnpA/B IS for chromosomal copy numbers 2–10. D) Sigmoid fits for all IS analyzed in this study and used to generate rate parameters in figure 1D. [file media-1.pdf]

A

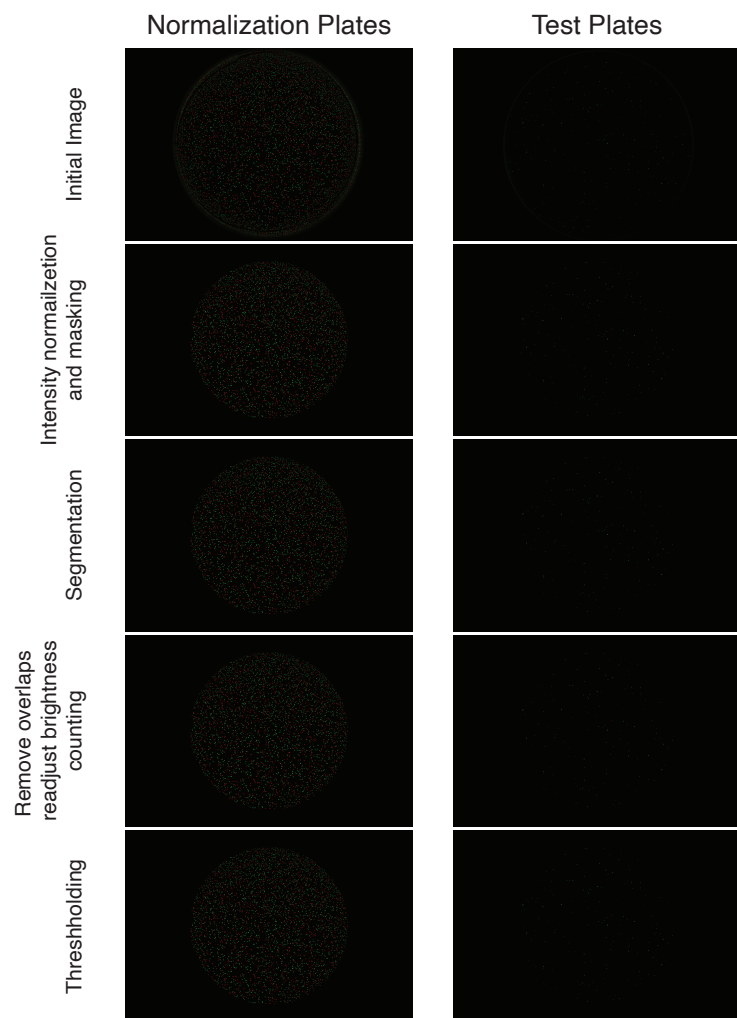

B

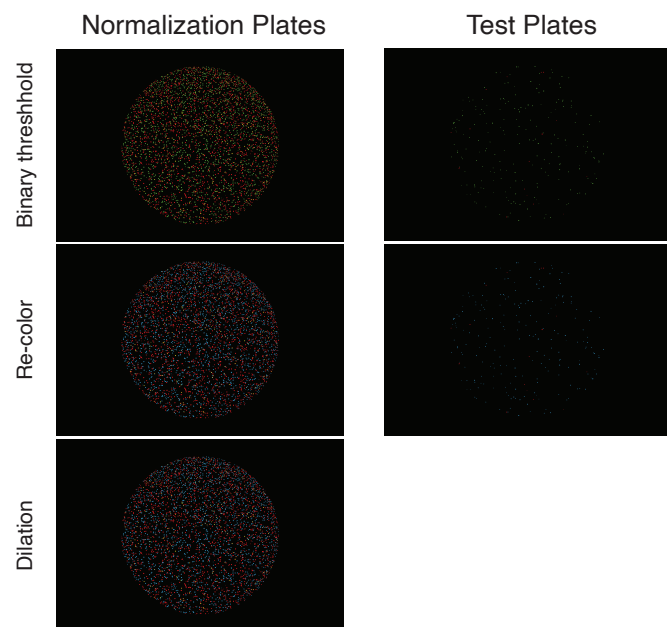

C

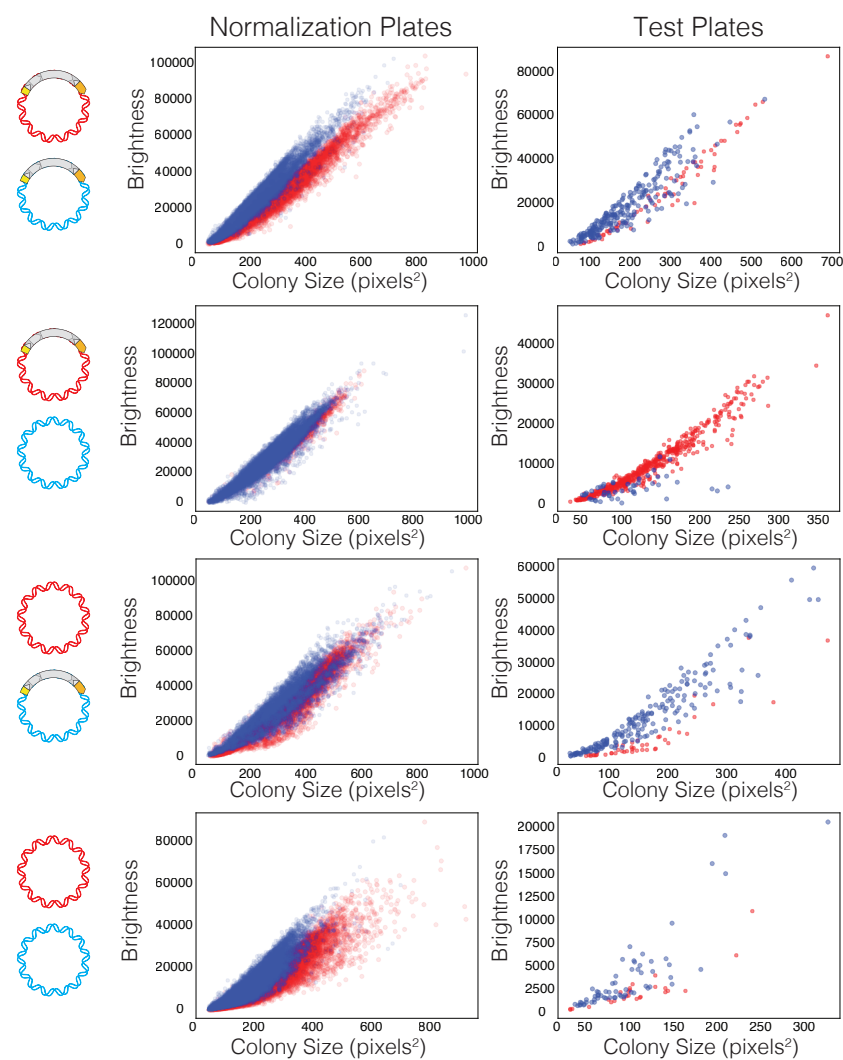

D

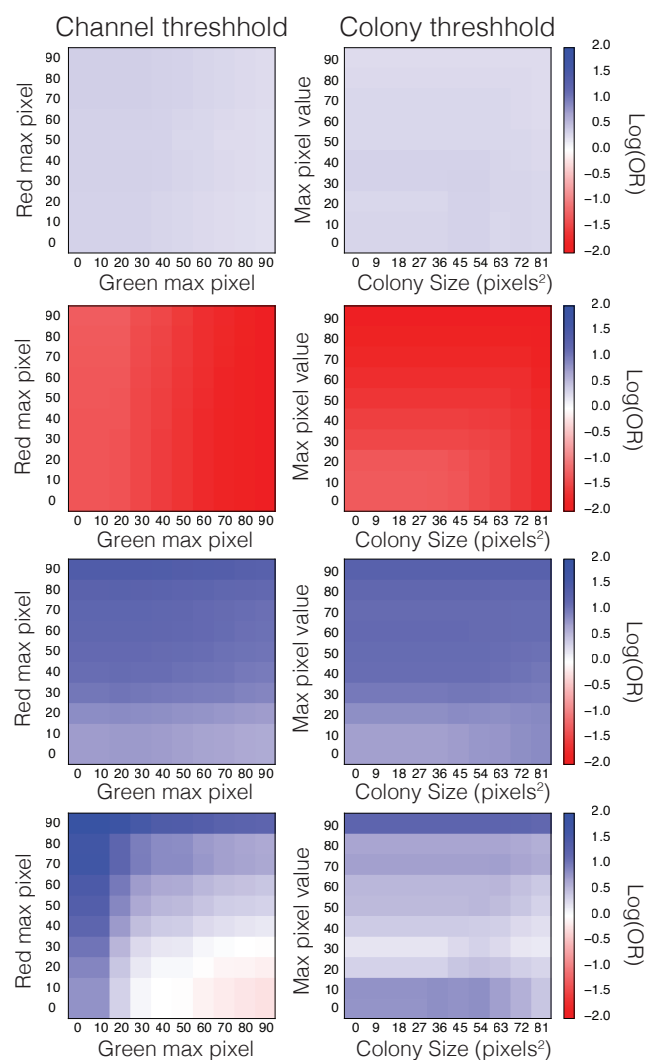

Supplement: Supplement 2 — Supplemental Figure 2: Imaging pipeline for superinfection assays A) Image workflow for colony counting. Overlapping colonies are only removed for test plates. B) image workflow for visualization. Binary threshold is imposed for colonies and the test colonies are dilated. C) Aggregate results prior to calculation of the log odds ratio. Plasmid conditions shown on the right in order top to bottom: both with IS, red with IS blue empty, blue with IS red empty, both empty. D) Threshold sensitivity analysis for the conditions in C for individual channel brightness thresholding and overall brightness and size thresholding. Tiling threshold difference in intensity between the fluorescence channels, and in intensity and colony size resulted in minimal deviation of the final log-odds ratio. There was slight sensitivity when competing two empty plasmids, where we saw a bias towards mWatermelon which was mirrored in our results. We attributed this bias to watermelon generally being a brighter fluorophore. [file media-2.pdf]
